# Common Shortcomings in Study on Radiopharmaceutical Design Research: A Case Study of ^99m^Tc-Labelled Methotrexate

**DOI:** 10.3390/molecules26195862

**Published:** 2021-09-27

**Authors:** Przemysław Koźmiński, Paweł Krzysztof Halik, Raphael Chesori, Ewa Gniazdowska

**Affiliations:** Centre of Radiochemistry and Nuclear Chemistry, Institute of Nuclear Chemistry and Technology, Dorodna 16, 03-195 Warsaw, Poland; p.halik@ichtj.waw.pl (P.K.H.); r.chesori@ichtj.waw.pl (R.C.); e.gniazdowska@ichtj.waw.pl (E.G.)

**Keywords:** methotrexate, radiopharmaceuticals, technetium-99m

## Abstract

The aim of the work carried out was to draw attention to shortcomings that often appear at the stage of designing new radiopharmaceuticals. Based on a case study of ^99m^Tc-labelled methotrexate, this article describes frequent mistakes or misconceptions present not only in the referenced studies, but also in numerous radiopharmaceutical studies. The recommendations provided in this article highlight fundamental aspects of the credibility of radiopharmaceutical scientific research leading to the reliable results.

## 1. Introduction

Scientific progress is nothing else than the process of improving the state of knowledge in a given field of science, carried out by making new scientific discoveries. In applied sciences such as medicine, the measure of scientific progress is more and more accurate explanation of the (physiological) processes and events as well as providing solutions to the problems related to these (pathological) phenomena that are of interest to this science. 

**Figure 1:**
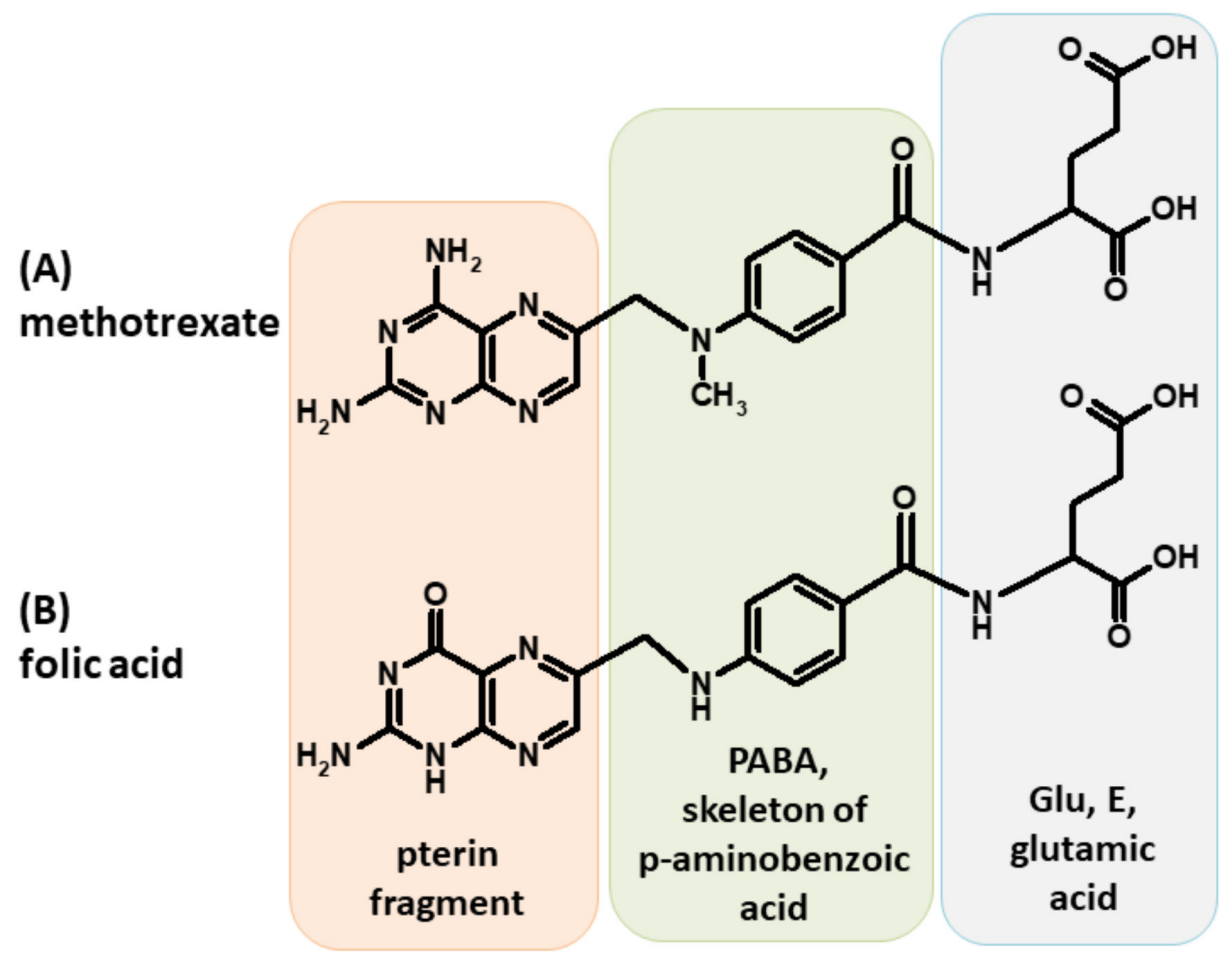
Molecular formulas of methotrexate (**A**) and folic acid (**B**) molecules.

Methotrexate (MTX, amethopterin) is widely characterised as an antineoplastic agent and one of the most extensively used disease-modifying anti-rheumatoid drugs belonging to the anti-folate family. MTX has been utilised for over half a century in oncological therapy in high doses against multiple types of tumours such as acute lymphoblastic and myelocytic leukaemia, choriocarcinoma, lung or breast cancer, and in low-dose therapy as a first-line drug in rheumatoid arthritis, or even in severe forms of psoriasis and chronic inflammatory diseases [1,2]. The MTX pharmacophore site responsible for its activity is considered as the reduced pterin fragment (Figure 1). In comparison to folic acid (FA), MTX affinity to folate receptors is noticeably lower due to replacement of oxygen atom in pterin moiety (present in FA molecule) with primary amino group [3].

MTX also has been labelled with diagnostic or therapeutic radionuclides (using auxiliary ligands in so-called pseudo-direct labelling method or specific bifunctional chelators) for needs of basic sciences and nuclear medicine applications [4]. Syntheses and studies of MTX radiocomplexes were also the subject of research conducted by our group. All issues and difficulties with pseudo-direct MTX labelling with technetium-99m, in accordance to the procedures described in the literature, forced us to analyse the conditions of the [^99m^Tc]Tc-MTX radiosynthesis in more detail.

Herein, we report our approach for pseudo-direct radiosynthesis of ^99m^Tc complexes on an example of MTX complex with technetium, [^99m^Tc]Tc-MTX. A brief overview on the perspectives of MTX application in nuclear medicine [4], as well as our own past laboratory experience, sensitised us to the reliance on others’ research that actually violated the accepted standards of scientific credibility. Current state of knowledge in the radiosyntheses of [^99m^Tc]Tc-MTX complex was found by us as unreliable and misleading. The present publication concerns the repeated syntheses of the [^99m^Tc]Tc-MTX complex according to published procedures, our approach to this radiochemical issue and its discussion, followed by recommendations and conclusion. We highlighted frequent mistakes or misconceptions present not only in referenced studies, but also in numerous radiopharmaceutical studies especially in commonly practiced ^99m^Tc radiolabelling procedures.

## 2. Methotrexate Labelling with Technetium-99m Case Study 

### 2.1. Direct Method of the Syntheses of [^99m^Tc]Tc-MTX Complex in Scientific Literature

The procedure of direct method of MTX labelling with technetium-99m one can find in several works [5,6,7,8,9,10,11,12]. Generally, the method is based on the reduction of ^99m^Tc^VII^ (from the pertechnetate anions, [^99m^Tc]TcO_4_^−^, obtained from ^99^Mo/^99m^Tc-generator in all below syntheses) to technetium-99m cation on a lower oxidation state, which allows the formation of [^99m^Tc]Tc-MTX complex. In this method as a reducing agent the cations of Sn^2+^ are used predominantly, however, the synthesis procedures of [^99m^Tc]Tc-MTX complex provided by different research groups often differ in the presence of various additional reagents.

The research of Jain et al. and Das et al. were aimed at labelling with technetium-99m the methotrexate-loaded stealth albumin nanoparticles (FA-PEG-HSA-MTX-NPs) [5] and the multiwalled carbon nanotube conjugated with methotrexate (AF-FA-MTX-MWCNTs) [7], to evaluate the effectiveness of application of these radiopreparation in scintigraphic imaging. In both articles [^99m^Tc]Tc-MTX complex was obtained as a comparative reference compound using direct labelling method. According to these reported methods free MTX, dissolved in minimum volume of 0.1 N HCl, was added into the mixture of sodium pertechnetate and appropriate amount of stannous chloride in 10% solution of acetic acid. The reaction mixture pH was adjusted to 6.5–7.0 using 0.5 M sodium bicarbonate solution. After 15 min of incubation at room temperature the radiolabelled formulation was separated from the radio colloids (reduced and hydrolysed ^99m^Tc in form of oxides) using Sephadex G-20 column [5] or by centrifugation [7]. The labelling efficiency of the purified [^99m^Tc]Tc-MTX complex was determined by instant thin layer chromatography (ITLC) using silica gel-coated fiber sheets and acetone as developing solution. Under this condition free [^99m^Tc]TcO_4_^−^ moved with the solvent (R_f_ ≈ 0.9), while the [^99m^Tc]Tc-MTX complex remained at the spotting point (R_f_ ≈ 0.1) [5]. It is worth emphasising that ITLC analysis was the only confirmation of the [^99m^Tc]Tc-MTX complex radiosynthesis (Table 1). 

Another report of direct MTX labelling with technetium-99m is described in the article of Dar et al. [6]. According to that, alkaline aqueous solution of MTX, ascorbic acid (AA), stannous tartrate and [^99m^Tc]NaTcO_4_ were incubated for 5 min at room temperature providing [^99m^Tc]Tc-MTX. Radiochemical purity of obtained radiocomplex was determined by two planar chromatography (PC) methods (Table 1). The authors also investigated the physicochemical properties of the obtained radiocomplex (stability and lipophilicity), but without providing specific information regarding whether these studies were performed on the radiopreparation previously isolated from the reaction mixture. 

Rasheed et al. synthesised and tested [^99m^Tc]Tc-MTX complex from the point of view of its potential application as a diagnostic agent for breast cancer imaging (within preliminary stage of clinical trial) [8] and as an imaging agent for an early rheumatoid arthritis nodules (within the first clinical trial) [13]. The authors synthesised [^99m^Tc]Tc-MTX complex according to the slightly modified procedure previously published by Dar et al. [6]. They conducted the synthesis of the radiocomplex using stannous tartrate additionally with AA, sodium citrate and pyrophosphate. All provided anions create complexes with technetium cations and act as the auxiliary ligands. After adjustment of pH to the value of 8.0–8.5, the mixture was incubated for 15 min at room temperature. The quality control of the labelling process was examined using PC methods in acetone or saline mobile solutions (Table 1). The authors also performed stability tests of [^99m^Tc]Tc-MTX complex, however as before, without information about any purification of the radiocomplex. Regarding lipophilicity assessment, there can only be found abstract information that “Partition coefficient in n-octanol and saline indicated that the labelled radiopharmaceutical was hydrophilic” [8]. Once more, these PC and ITLC analyses were the only confirmation of the reliability of the [^99m^Tc]Tc-MTX complex obtainment.

Singh and Singh synthesised and tested ^99m^Tc-radiocomplexes of MTX and MTX derivatives with glutamic acid, MTX-Glu_2_, [9] and lysine, MTX-Lys_2_, [10]. Amino acids Glu and Lys were coupled with MTX molecule via the nitrogen atoms of the pharmacophore pterin fragment in order to facilitate the MTX blood–brain barrier crossing and thereby delivery to the brain. In the direct labelling method, the reaction mixture of MTX or its derivative, [^99m^Tc]NaTcO_4_ and SnCl_2_ in 1 N HCl was neutralised using 0.5 M sodium bicarbonate solution and incubated at 25 ± 5 °C for 15–20 min. Further, radiolabelling efficiency was determined by the ITLC method using silica gel strips as stationary phase in combination with acetone or individually selected mobile phase (Table 1). The authors performed several stability tests (in PBS buffer pH 7.4 and saline solution) for each [^99m^Tc]Tc-MTX, [^99m^Tc]Tc-MTX-Glu_2_ and [^99m^Tc]Tc-MTX-Lys_2_ complex. Nevertheless, the only physicochemical parameters characterising the radiocomplexes obtained in the work were the results of ITLC analyses (Table 1). 

Ozgenc et al. published a simple MTX radiolabelling method with technetium-99m and investigated [^99m^Tc]Tc-MTX complex as a potential diagnostic radiopharmaceutical for breast cancer imaging [11]. Their “simple, rapid and efficient direct method for labelling of MTX with ^99m^Tc” was performed using stannous tartrate with AA to minimise the auto-radiolysis phenomenon. The pH value of reaction mixture was adjusted to pH 9 and the mixture was incubated at room temperature for 5 min. The radiochemical purity and stability of [^99m^Tc]Tc-MTX were assessed again only through TLC analyses (Table 1). The authors also performed stability tests of obtained [^99m^Tc]Tc-MTX complex in saline and human serum, however, in both cases tests were performed using aliquots from the labelling reaction mixture.

Papachristou et al. studied the possible use of ^99m^Tc-labelled MTX as a radiotracer for the identification of chemically induced inflammatory sites [12]. The labelling reaction of MTX was performed in neutral pH, in presence of sodium gluconate, sodium bicarbonate and stannous chloride at room temperature for 30 min. The authors indicated that the target radiocomplex was formed through the ligand exchange reaction from a [^99m^Tc]Tc^(V)^O-gluconate precursor. Radiochemical control of [^99m^Tc]Tc-MTX complex was carried out using two different PC methods executed in acetone and saline (Table 1). Furthermore, in the work are also presented the results of stability and lipophilicity studies (Table 1), but unfortunately, the authors did not provide information whether the complex was previously isolated from the reaction mixture. As the only ones, they also characterised [^99m^Tc]Tc-MTX complex using HPLC method and on the recorded radiochromatogram they obtained one product peak with the retention time (R_T_) of approximately 20 min, while R_T_ values of [^99m^Tc]TcO_4_^−^ and [^99m^Tc]Tc^(V)^O-gluconate precursor were <5 min; however, HPLC-radiochromatogram is not included in the article.

Concise information concerning above-described methods of MTX labelling with technetium-99m are presented in Table 1.

As it can be seen, in practically all the cited articles, the only verification of [^99m^Tc]Tc-MTX complex syntheses were the TLC analyses. Only in the Papachristou’s report [12], there is information that [^99m^Tc]Tc-MTX complex was formed as a result of ligand exchange reaction from the intermediate complex [^99m^Tc]Tc^(V)^O-gluconate precursor. Nevertheless in most reports (except [9,10]) in the reaction mixtures of [^99m^Tc]Tc-MTX complex synthesis, other ligands (acetate, tartrate, ascorbate, citrate and gluconate ions) capable of complexing technetium cations were present. To explain the course of reported above syntheses of the [^99m^Tc]Tc-MTX complex, incorrectly named as direct methods, we investigated physicochemical properties of various intermediate ^99m^Tc complexes that could be formed between ^99m^Tc and auxiliary ligands applied in these reactions. The physicochemical characterisation of intermediate ^99m^Tc complexes and final [^99m^Tc]Tc-MTX complex will allow to determine which radiocomplexes are formed during the ^99m^Tc-labelling procedures in the above-described conditions.

### 2.2. Direct Methods of Syntheses of the [^99m^Tc]Tc-Intermediate Complexes in Scientific Literature

**Scheme 1:**
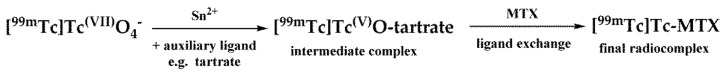
Scheme of two-step synthesis of the [^99m^Tc]Tc-MTX radiocomplex.

We searched the literature for reports on the preparation, testing and application in nuclear medicine or other fields of science, of molecules that are able to directly chelate reduced technetium-99m cations and can act as the auxiliary ligands in two-step syntheses of the desired final ^99m^Tc-radiopreparations (Scheme 1).

There are several anions that meet this requirement as citrates, ascorbates, various hydroxy acids and other similar compounds. These compounds labelled with technetium-99m were described in a few reports [14,15,16,17,18,19,20,21,22,23,24], but unfortunately, only in some of them the authors provide the preparation procedures and results of physicochemical tests for obtained ^99m^Tc-radiocomplexes [15,17,18,21,22,23,24] (Table 2).

Baker et al. examined EDTA and its analogues labelled with ^99m^Tc into the potential scintigraphic applications [15]. EDTA solution in saline was mixed with SnCl_2_ solution, followed by addition of [^99m^Tc]NaTcO_4_ in saline up to 60 min at room temperature. Reactants were carried out at pH 5.0 and thoroughly purged with nitrogen. Labelling efficiency evaluation was performed through cellulose acetate electrophoresis, additionally verified by ITLC analyses resolved in four systems. 

Ercan et al. studied [^99m^Tc]Tc-citrate as an inflammatory imaging agent and compared the effectiveness of inflammation sites imaging with that using [^67^Ga]Ga-citrate [17,18]. [^99m^Tc]Tc-citrate was synthesised at pH 5 at room temperature for 10 min using citric acid, sodium pertechnetate and stannous chloride as a reducing agent. The authors determined labelling efficiency by PC (Table 2), performed an electrophoretic analysis ([^99m^Tc]Tc-citrate creates a neutral complex) and plasma stability assay ([^99m^Tc]Tc-citrate was found to be stable and bound to plasma proteins only to a negligible extent). The percentage of radioactivity extracted from saline phase into the n-butanol phase (0.343 ± 0.107%) allowed to evaluate radiocomplex lipophilicity. However, there is no information in both articles whether these tests were performed on the isolated radiocomplex, and it can rather be assumed that all tests were conducted on the aliquots from the reaction mixture.

The procedure for labelling of AA with technetium-99m and basic physicochemical tests of the obtained radiocomplex can be found in the reports of Yigit et al. and Mamede et al. [21,22]. For labelling reaction Yigit et al. [21] applied AA dissolved in pure water, SnCl_2_ in 0.1 N HCI, and [^99m^Tc]NaTcO_4_. Labelling reaction was carried out at pH 5 at 25 °C for 20 min. Reaction progress and radiochemical purity of obtained [^99m^Tc]Tc-AA were evaluated through TLC and HPLC methods [21] (Table 2). The authors also performed an electrophoresis assay ([^99m^Tc]Tc-AA creates a negatively charged complex) and stability in human serum, however both tests were conducted on unpurified samples from the reaction mixture, what doubtlessly had an impact on determined [^99m^Tc]Tc-AA complex characteristics. A different labelling procedure of AA with technetium-99m is described by Mamede et al. [22]. The authors conducted reaction of sodium pertechnetate, AA and iron(III) chloride at room temperature at pH 6.5–7 in argon atmosphere and protected from light. Determination of R_T_ values for all applied reagents and identification of the final [^99m^Tc]Tc-AA complex were realised using HPLC method (Table 2), similarly as further determination of radiochemical purity of [^99m^Tc]Tc-AA complex. Comparison of the radiochromatograms recorded separately for each reactant allowed for the identification of all components (applied substrates and obtained products) present in the reaction mixture.

Park et al. labelled with technetium-99m various α-hydroxy acids, such as glycolate, L-lactate, D-lactate, D-gluconate, D-glucoheptonate, D-glucuronate, D-glucarate and citrate (β-hydroxy acid) [23]. The aim of their research was to develop a ^99m^Tc-labelled agent for in vivo imaging of endogenous hydrogen sulfide, an important mediator of physiological function of gastrointestinal tract. Labelling reactions of α-hydroxy acids were carried out using a specific α-hydroxy acid, sodium pertechnetate and stannous chloride at pH 6.5 at room temperature for 20 min. All labelled products were analysed only by PC method (Table 2).

Concise information concerning described above syntheses of the ^99m^Tc-complexes are presented in Table 2.

**Figure 2:**
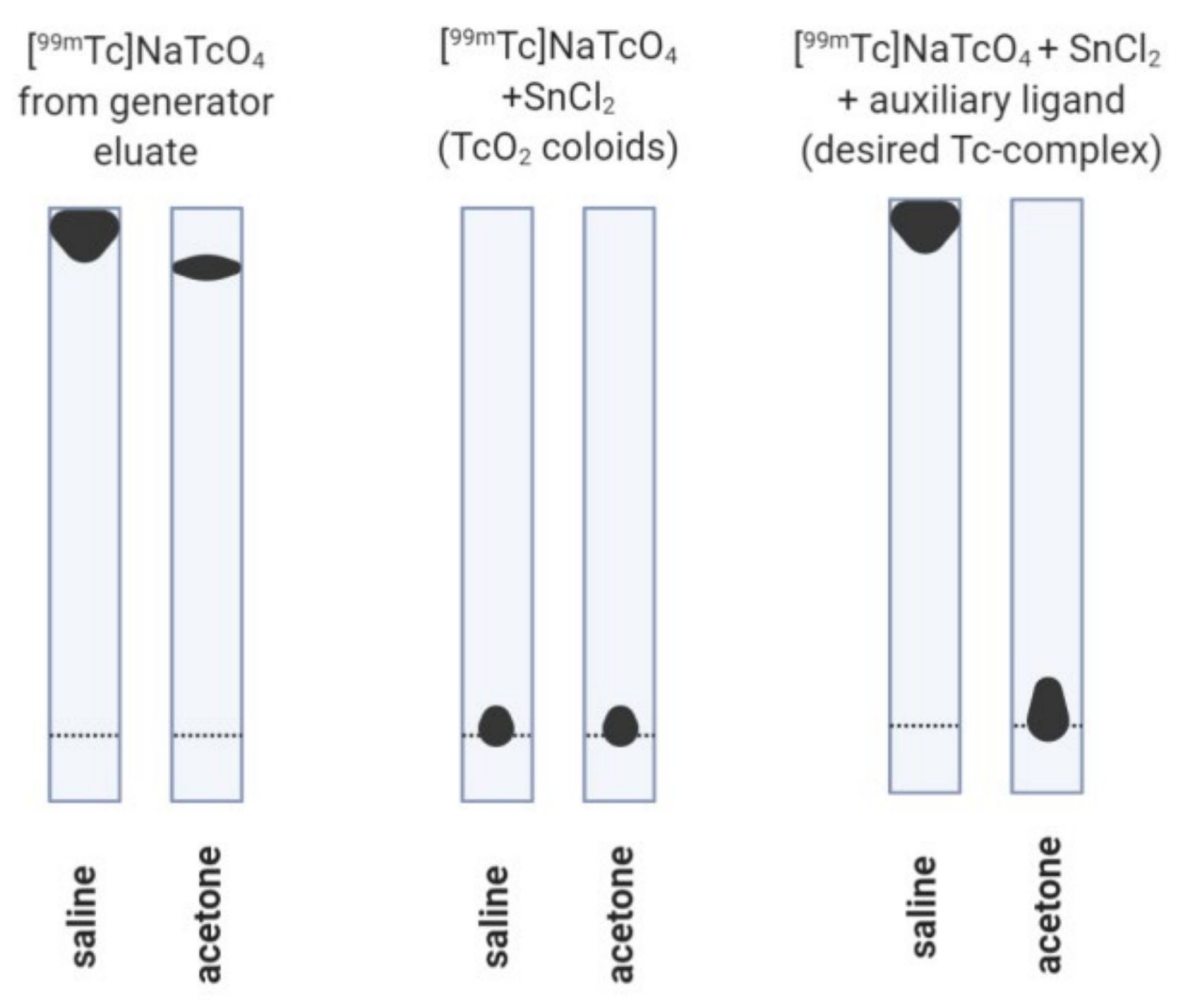
Schematic results of TLC analysis developed in saline (aqueous) and acetone (organic) mobile phases of free [^99m^Tc]NaTcO_4_ (left two strips), reduced pertechnetate in form of [^99m^Tc]TcO_2_ (middle two strips) or ^99m^Tc-complex of molecule that are able to directly chelate reduced ^99m^Tc cations (right two strips).

As one can see in Table 2, PC was the most common method for identification of the labelling reaction products described above. All obtained radiocomplexes had the same analytical profile—they moved with the solvent front in the case of the saline or ACD solution mobile phase and remained at the spotting point in the case of acetone or serum physiologic (Figure 2).

### 2.3. Own Results of Syntheses of the ^99m^Tc-Intermediate Complexes and the Final [^99m^Tc]Tc-MTX Complex

#### 2.3.1. [^99m^Tc]Tc-MTX Radiocomplex Synthesis

We initiated our research from repetition of the procedures described in the cited articles on MTX radiolabelling with ^99m^Tc. Like other research groups (Table 1), we performed the MTX radiolabelling reaction without or with various additional reagents (auxiliary ligands) in similar manner as it was performed in articles referred in Table 1. In parallel, we performed the separate syntheses of intermediate ^99m^Tc complexes with applied auxiliary ligands. We also adopted a terminology consistent with the real course of the labelling reaction, namely for the MTX labelling reaction without auxiliary ligands we adopted the term direct method, while in the presence of auxiliary ligands—the indirect or pseudo-direct method.

[^99m^Tc]Tc-MTX synthesis without auxiliary ligands: First, synthetic experiments were conducted according to direct labelling method. In total, 5 mg of MTX were dissolved in deionised water alkalised with 1M NaOH to pH 9. Into this solution, 1 mL of 100–500 MBq of [^99m^Tc]NaTcO_4_ and 50 µL of fresh solution containing 100 µg of stannous chloride in 0.1M HCl. Reaction mixture was incubated for 30 min at room temperature followed by ITLC and HPLC analyses, that showed no reaction occurred. 

**Figure 3:**
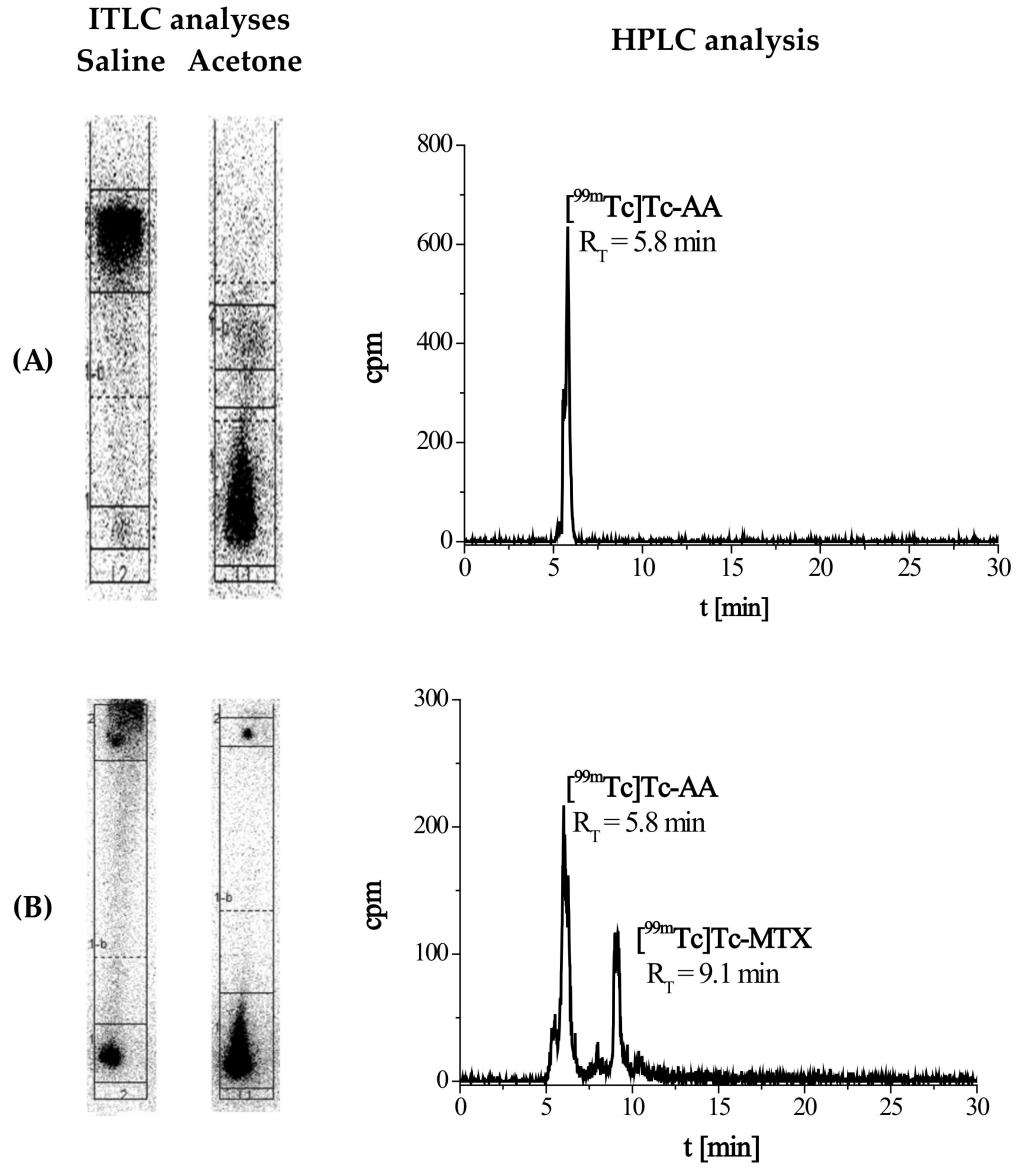
ITLC and HPLC analyses verifying formation of (**A**) intermediate [^99m^Tc]Tc-AA complex and (**B**) final [^99m^Tc]Tc-MTX complex. Basing on ITLC results, differentiation between final radiocomplex and intermediate radiocomplex is impossible without specific aid in form of HPLC analysis. Only then, it can be assessed that formation of [^99m^Tc]Tc-MTX complex occurred to a small extent.

[^99m^Tc]Tc-MTX synthesis in the presence of AA: Before the synthesis of [^99m^Tc]Tc-MTX complex, we performed the synthesis of the intermediate [^99m^Tc]Tc-AA complex as follows: into 1 mg of AA dissolved in 0.2 mL of saline, 1 mL of 100–500 MBq of [^99m^Tc]NaTcO_4_ and 50 µL of fresh solution containing 100 µg of stannous chloride in 0.1M HCl. The final pH 6–7 was adjusted with 1M NaOH and the reaction mixture was incubated for 5 min at room temperature, followed by ITLC and HPLC analyses, presented below in Figure 3A. The yield of radiocomplex creation was determined as higher than 95% (based on TLC analysis in reaction mixture only trace amounts of [^99m^Tc]Tc-radiocolloids and pertechnetate anions were visible).

Similarly, into 1 mg of AA and 2 mg of MTX in alkaline aqueous solution (pH 9), 1 mL of 100–500 MBq of [^99m^Tc]NaTcO_4_ and 50 µL of fresh solution containing 100 µg of stannous chloride in 0.1 M HCl were added. After incubation of reaction mixture for 30 min at room temperature, ITLC and HPLC analyses were performed (Figure 3B). Based on these analyses one can say that three radiospecies were formed during the radiolabelling reaction: [^99m^Tc]Tc-radiocolloids—visible only in ITLC analyses (during HPLC analysis these species remained permanently bound on the HPLC pre-column), [^99m^Tc]Tc-AA and [^99m^Tc]Tc-MTX radiocompounds. Evaluation of the labelling reaction efficiency (based on TLC and HPLC analysis) showed that the yield of [^99m^Tc]Tc-MTX radiocomplex formation was about 11% ([^99m^Tc]Tc-radiocolloids were formed with the yield about 62%, [^99m^Tc]Tc-AA—about 24% and about 3% of the radioactivity remained in the form of [^99m^Tc]TcO_4_^-–^ unreduced anions).

**Figure 4:**
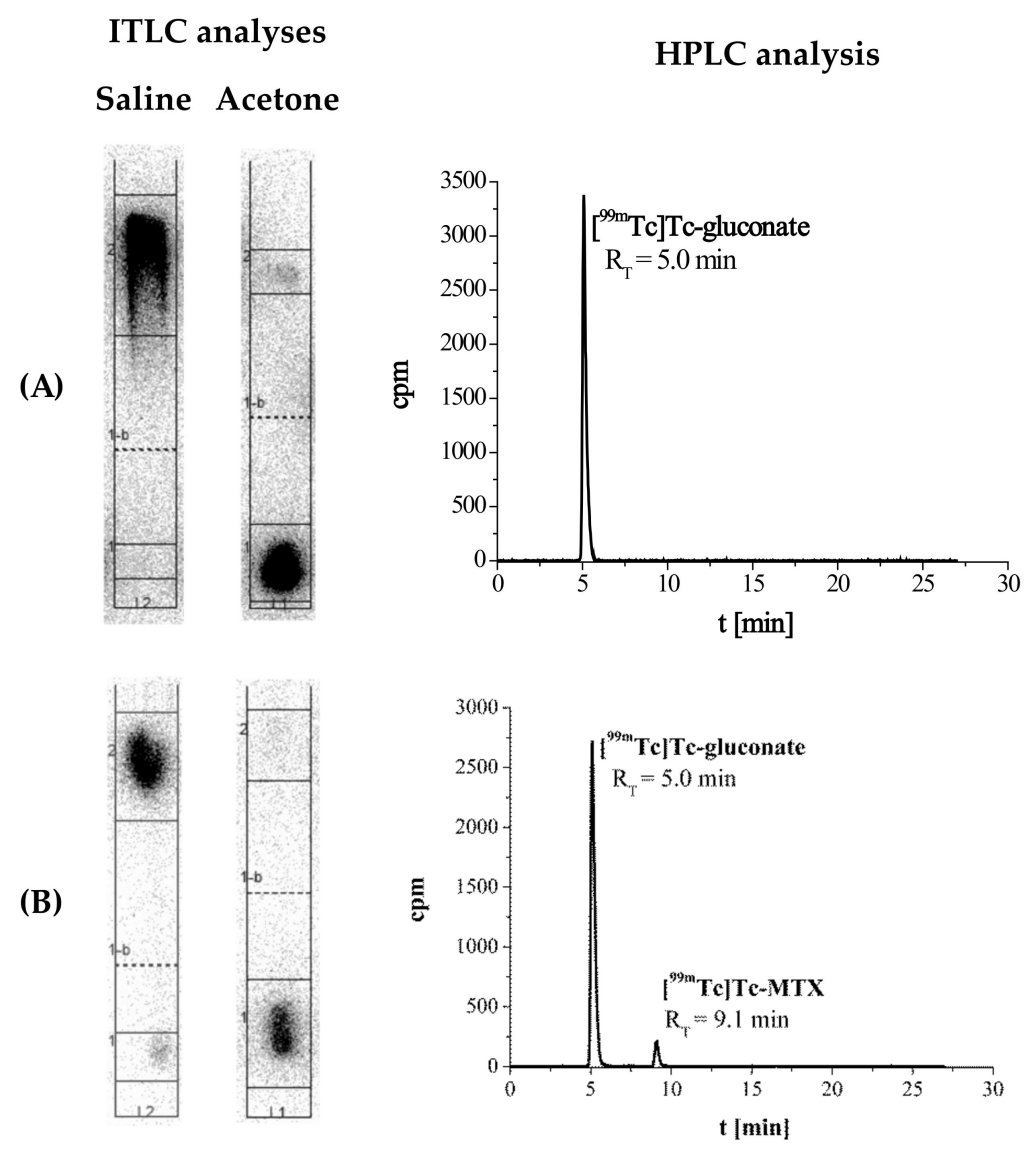
ITLC and HPLC analyses verifying formation of (**A**) intermediate [^99m^Tc]Tc-gluconate complex and (**B**) final [^99m^Tc]Tc-MTX complex. Basing on ITLC results, differentiation between final radiocomplex and intermediate radiocomplex is impossible without specific aid in form of HPLC analysis. Only then, it can be assessed that formation of [^99m^Tc]Tc-MTX complex occurred to a negligible extent.

[^99m^Tc]Tc-MTX synthesis in the presence of sodium gluconate: The synthesis of the intermediate [^99m^Tc]Tc-gluconate complex was performed as follows: into 1 g of sodium gluconate, 1 mL of 100–500 MBq of [^99m^Tc]NaTcO_4_ eluate and 50 µL of fresh solution containing 100 µg of stannous chloride in 0.1M HCl were added for 5 min incubation at room temperature. Radiochemical yield of the intermediate [^99m^Tc]Tc-gluconate complex was analysed through ITLC and HPLC methods that provide clear assessment on complex creation, presented below in Figure 4A. The yield of radiocomplex creation was determined as higher than 98%.

Similarly, into 1 g of sodium gluconate and 10 mg of MTX in alkaline aqueous solution (pH 9), 1 mL of 100–500 MBq of [^99m^Tc]NaTcO_4_ and 50 µL of fresh solution containing 100 µg of stannous chloride in 0.1M HCl were added. After incubation of reaction mixture for 30 min at room temperature, ITLC and HPLC analyses were performed (Figure 4B). The yield of [^99m^Tc]Tc-MTX radiocomplex formation in this radiolabelling reaction was determined as 12%. There were practically no [^99m^Tc]Tc-radiocolloids and [^99m^Tc]TcO_4_^-–^ anions present in both reaction mixtures.

**Figure 5:**
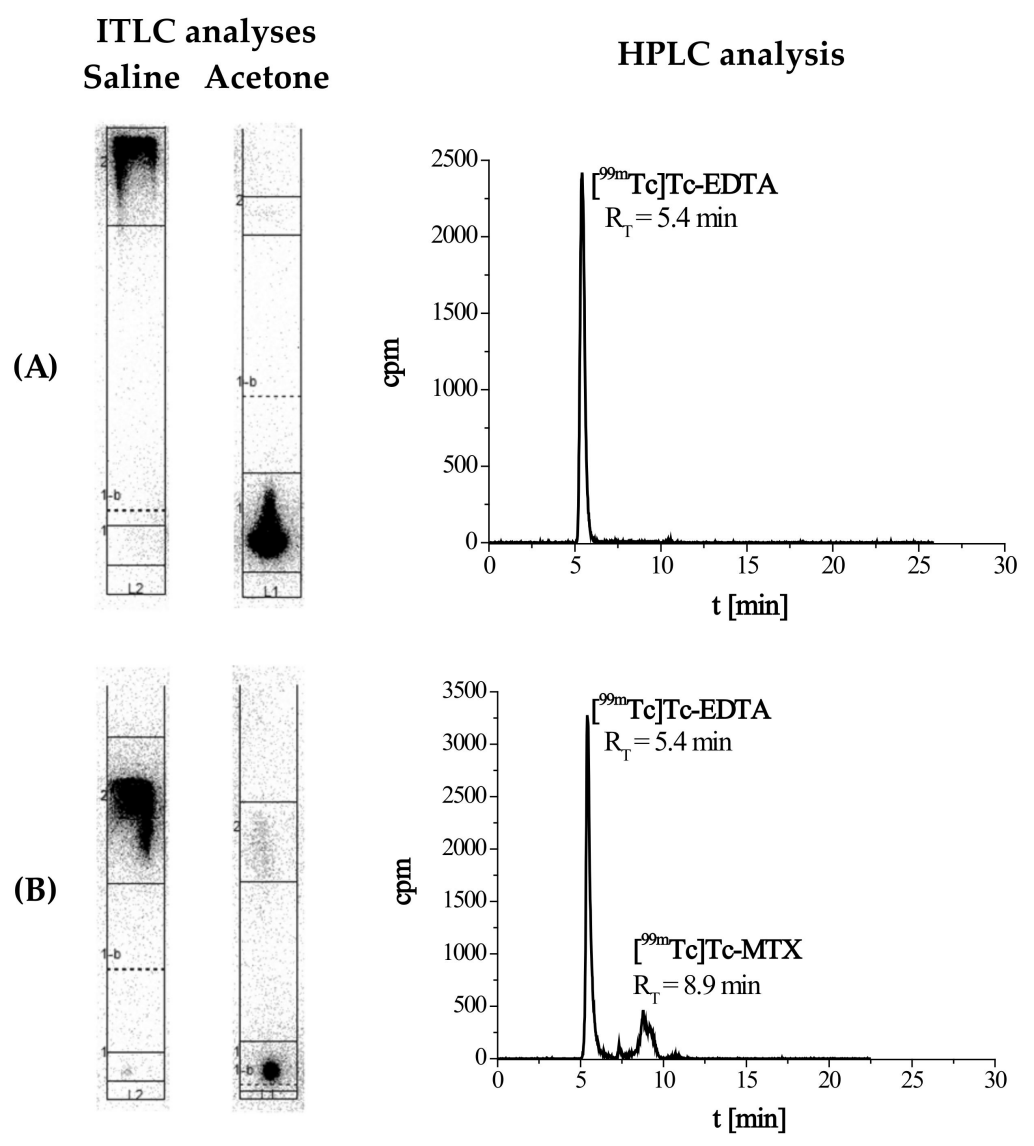
TLC and HPLC analyses verifying formation of (**A**) intermediate [^99m^Tc]Tc-EDTA complex and (**B**) final [^99m^Tc]Tc-MTX complex. Basing on ITLC results, differentiation between final radiocomplex and intermediate radiocomplex is impossible without specific aid in form of HPLC analysis. Only then, it can be assessed that formation of [^99m^Tc]Tc-MTX complex occurred to a negligible extent.

[^99m^Tc]Tc-MTX synthesis in the presence of EDTA: The synthesis of the intermediate [^99m^Tc]Tc-EDTA complex was performed as follows: into 5 mg of EDTA and 5 mg of mannitol, 1 mL of 100–500 MBq of [^99m^Tc]NaTcO_4_ eluate and 50 µL of fresh solution containing 100 µg of stannous chloride in 0.1M HCl were added for 5 min incubation at room temperature. Radiochemical yield of the intermediate [^99m^Tc]Tc-EDTA complex was analysed through ITLC and HPLC methods that provide clear assessment on complex creation, presented below in Figure 5A. The yield of intermediate [^99m^Tc]Tc-EDTA radiocomplex creation was determined as higher than 98%.

Similarly, into 5 mg of EDTA, 5 mg of mannitol and 11 mg of MTX in alkaline aqueous solution (pH 9), 1 mL of 100–500 MBq of [^99m^Tc]NaTcO_4_ and 50 µL of fresh solution containing 100 µg of stannous chloride in 0.1M HCl were added. After incubation of reaction mixture for 30 min at room temperature, ITLC and HPLC analyses were performed (Figure 5B). The yield of [^99m^Tc]Tc-MTX radiocomplex creation was determined as 23% ([^99m^Tc]Tc-EDTA was formed with the yield about 77%). As in the previous case, there were no [^99m^Tc]Tc-radiocolloids and [^99m^Tc]TcO_4_^−^ anions present in both reaction mixtures.

**Figure 6:**
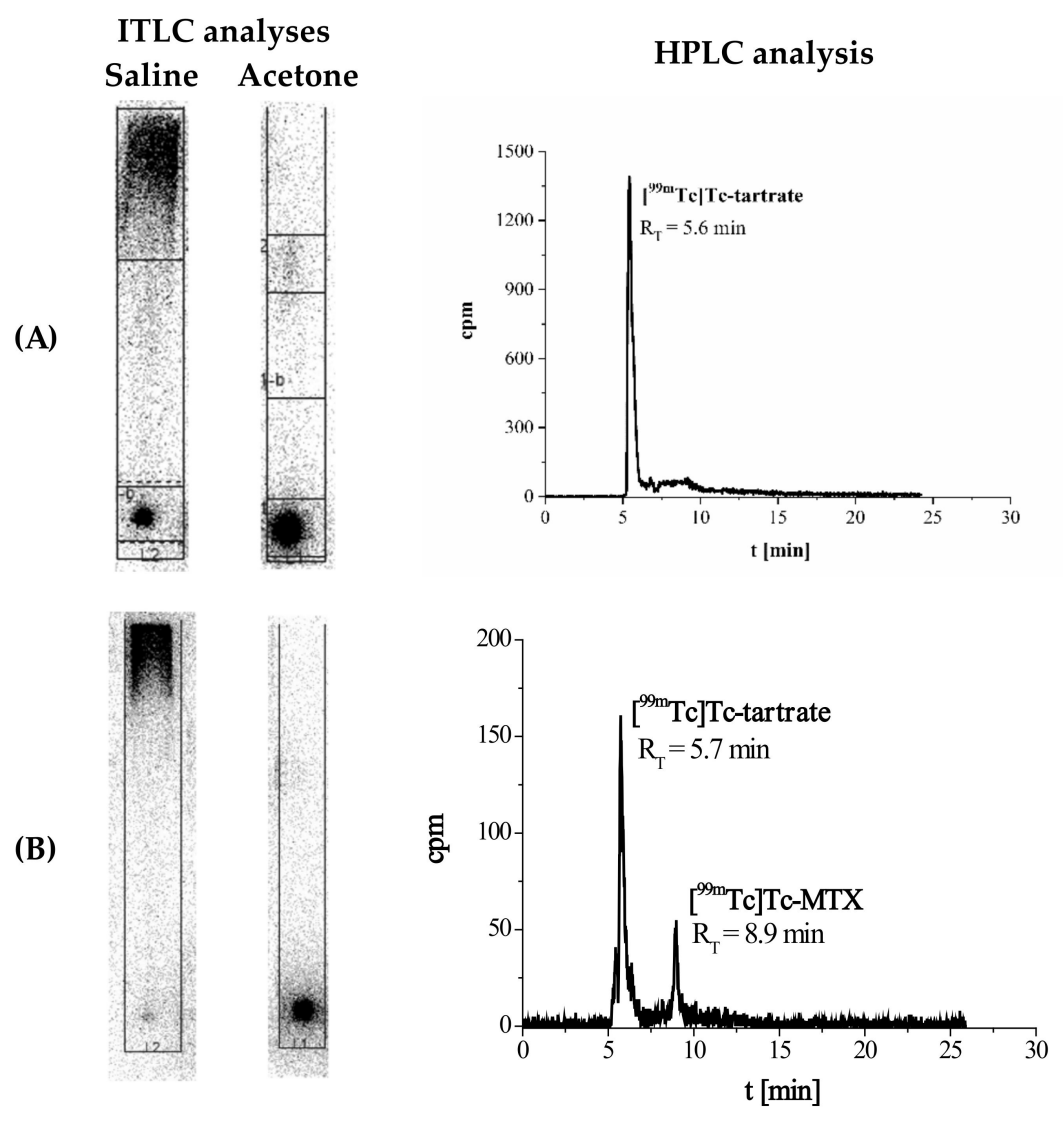
ITLC and HPLC analyses verifying formation of (**A**) intermediate [^99m^Tc]Tc-tartrate complex and (**B**) final [^99m^Tc]Tc-MTX complex. Basing on ITLC results, differentiation between final radiocomplex and intermediate radiocomplex is impossible without specific aid in form of HPLC analysis. Only then, it can be assessed that formation of [^99m^Tc]Tc-MTX complex occurred to a small extent.

[^99m^Tc]Tc-MTX synthesis in the presence of potassium tartrate: The synthesis of the intermediate [^99m^Tc]Tc-tartrate complex was performed as follows: into 1.1 mg of potassium tartrate, 1 mL of 100–500 MBq of [^99m^Tc]NaTcO_4_ eluate and 50 µL of fresh solution containing 100 µg of stannous chloride in 0.1M HCl were added for 5 min incubation at room temperature. Radiochemical yield of the intermediate [^99m^Tc]Tc-tartrate complex was analysed through ITLC and HPLC methods that provide clear assessment on complex creation, presented below in Figure 6A. In this case, ITLC and HPLC analyses showed the formation of two radiospecies: [^99m^Tc]Tc-radiocolloids (not visible in the HPLC chromatograms) and [^99m^Tc]Tc-tartrate radiocomplex. Based on these analyses the yield of [^99m^Tc]Tc-radiocolloids creation was determined as about 27%, and the yield of intermediate [^99m^Tc]Tc-tartrate radiocomplex as 73%.

Similarly, into 1.1 mg of potassium tartrate and 5 mg of MTX in alkaline aqueous solution (pH 9), 1 mL of 100–500 MBq of [^99m^Tc]NaTcO_4_ and 50 µL of fresh solution containing 100 µg of stannous chloride in 0.1M HCl were added. After incubation of reaction mixture for 5 min at room temperature, ITLC and HPLC analyses were performed (Figure 6B). Based on HPLC analysis the yield of [^99m^Tc]Tc-MTX complex formation was determined as about 39% and the yield of intermediate [^99m^Tc]Tc-tartrate radiocomplex as about 61%. There were no [^99m^Tc]TcO_4_^-–^ anions present in the labelling reaction mixture and the amounts of [^99m^Tc]Tc-radiocolloids were traceable.

The conditions of above reactions were taken from the cited scientific articles in Table 1, however, a synthesis reaction that uses [^99m^Tc]Tc-tartrate intermediate complex we optimised in order to receive desired [^99m^Tc]Tc-MTX complex more efficiently for basic physicochemical characterisation.

In the first step, the intermediate complex synthesis was optimised in terms of applied amount of potassium tartrate in range 0.22–1.32 mg (Table 3). The most optimal creation of intermediate complex took place above 1 mg of substrate, therefore the synthesis of [^99m^Tc]Tc-tartrate complex was performed as follows: into 1.1 mg of potassium tartrate, 1 mL of 100–500 MBq of [^99m^Tc]NaTcO_4_ eluate and 50 µL of fresh solution containing 100 µg of stannous chloride in 0.1M HCl were added for 5 min incubation at room temperature. The results of the ITLC and HPLC analyses showed high radiochemical yield of [^99m^Tc]Tc-tartrate complex formation and were very similar to those presented above in Figure 6A.

**Figure 7:**
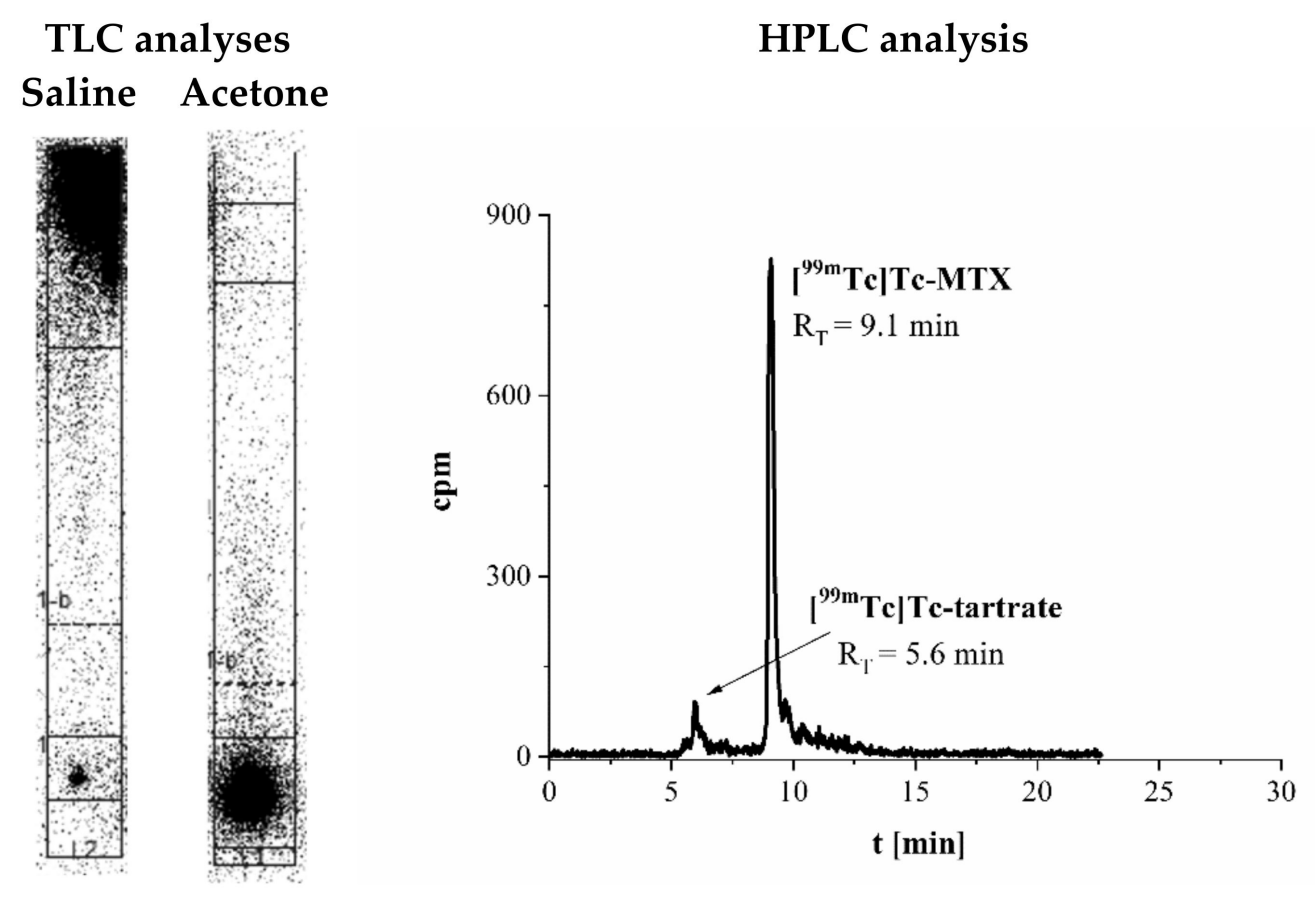
ITLC and HPLC analyses verifying formation of final [^99m^Tc]Tc-MTX complex after 120 min. Basing on ITLC results, differentiation between final radiocomplex and intermediate radiocomplex is impossible without specific aid in form of HPLC analysis. Only then, it can be assessed that formation of [^99m^Tc]Tc-MTX complex reached a satisfactory degree.

In the second step, the synthesis of the [^99m^Tc]Tc-MTX radiocomplex was evaluated in relation to various amounts of MTX in range of 1–10 mg (Table 4). The highest yield was obtained for 5 mg MTX in alkaline aqueous solution (pH 9) and 1.1 mg of potassium tartrate mixed with 1 mL of 100–500 MBq of [^99m^Tc]NaTcO_4_ and 50 µL of fresh solution containing 100 µg of stannous chloride in 0.1 M HCl. Then, reaction mixture was purged with nitrogen and incubated at room temperature up to 120 min, followed by ITLC and HPLC analyses, presented below in Figure 7.

#### 2.3.2. Properties Determination of ^99m^Tc-Intermediate and [^99m^Tc]Tc-MTX Complexes

**Figure 8:**
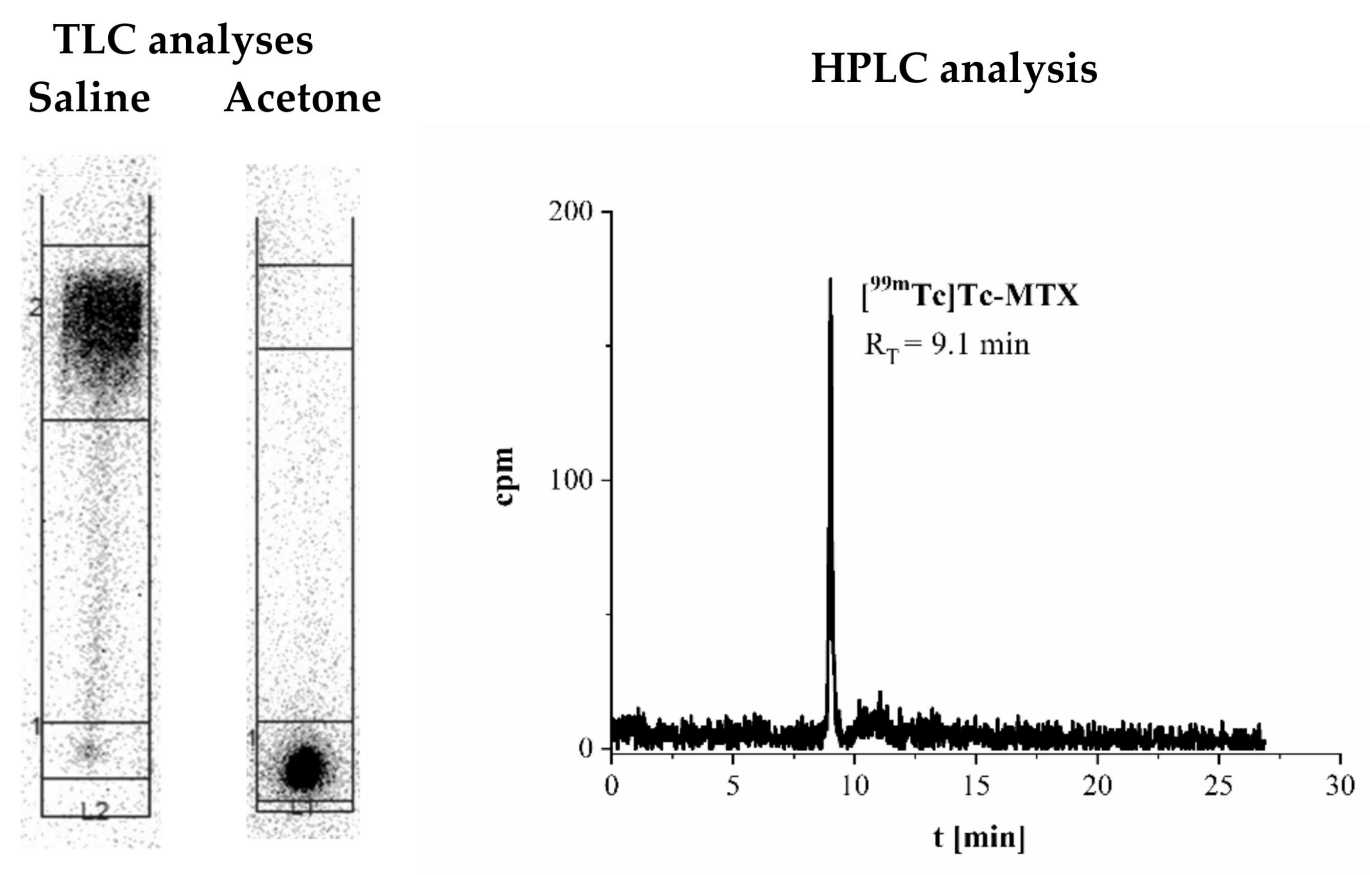
ITLC and HPLC analyses verifying stability of isolated [^99m^Tc]Tc-MTX complex during the lipophilicity study.

In all ^99m^Tc-intermediate complex syntheses the obtained radiocomplex was isolated from the reaction mixture using HPLC in order to perform TLC analyses and lipophilicity determination (Table 5). In similar manner, TLC analyses and lipophilicity determination were performed for [^99m^Tc]Tc-MTX complex isolated from reaction mixture and as aliquot from synthesis reaction mixture. At the end of the lipophilicity test, ITLC and HPLC analyses of the aqueous phase were performed to verify the stability of radiopreparation of interest during the experiment period. The chromatogram of aqueous phase with [^99m^Tc]Tc-MTX complex is presented below in Figure 8.

#### 2.3.3. Discussion of Results

Supported by a literature review, in all our syntheses, we obtained desired ^99m^Tc-intermediate complex, what was confirmed through HPLC method. The first attempt of [^99m^Tc]Tc-MTX complex synthesis according to described in literature direct labelling method finished in failure, however, further efforts led to creation of a desired radiocomplex through indirect labelling method using intermediate [^99m^Tc]Tc-tartrate complex with high satisfactory yield. On the other hand, reactions performed according to the procedures given by the authors of the cited reports provided poor outcome of [^99m^Tc]Tc-MTX complex formation, practically in trace amounts. Moreover, the authors claimed that they performed direct labelling of MTX with ^99m^Tc radionuclide, despite application of additional reagents that are able to directly chelate reduced ^99m^Tc cations. Generally, ^99m^Tc labelling occurs as one- or two-step radiosynthesis, defined as direct or indirect methods. In the direct labelling method, the reduction of Tc^(VII)^O_4_^−^ to temporary Tc^(V)^ form by Sn^2+^ cations in deoxygenated conditions, is followed by instant formation of the target complex. In the indirect method, reduced technetium first creates an intermediate complex with an auxiliary ligand, that stabilises the Tc^(V)^ oxidation stage, to finally participate in ligand exchange reaction leading to the conversion of the auxiliary ligand anions to the target anions (Scheme 1) [23,25,26,27]. Indirect radiolabelling method is applied in order to label ligands with poor solubility in aqueous solutions or whether the radiolabelling has a low reaction rate [25].

Research discussed above (Table 1) was based on TLC evaluation of the course and yield of the labelling reaction, while only in one study HPLC analysis was performed [12], although without including any radiochromatogram. In response to these, we provided the results of TLC analyses carried out for the syntheses of corresponding intermediate complexes. As one can see, the results of the TLC analyses are always the same—^99m^Tc-complexes migrate with the front of saline mobile phase (R_f_ ≈ 1) and stay at the origin in acetone (R_f_ ≈ 0) (Table 5). Thus, it is impossible to distinguish any ^99m^Tc-intermediate complex and the [^99m^Tc]Tc-MTX complex basing only on the results of TLC analyses. Based on that, we consider that in any case of the research cited in Table 1 [5,6,7,8,9,10,11,12], it was not possible to distinguish between the ^99m^Tc-intermediate complexes ([^99m^Tc]Tc-AA, [^99m^Tc]Tc-tartrate, [^99m^Tc]Tc-citrate, [^99m^Tc]Tc-gluconate) from the final product [^99m^Tc]Tc-MTX complex using only PC method. All ^99m^Tc-intermediate complexes as well as [^99m^Tc]Tc-MTX radiocomplex in TLC analyses behaved in a similar manner.

**Figure 9:**
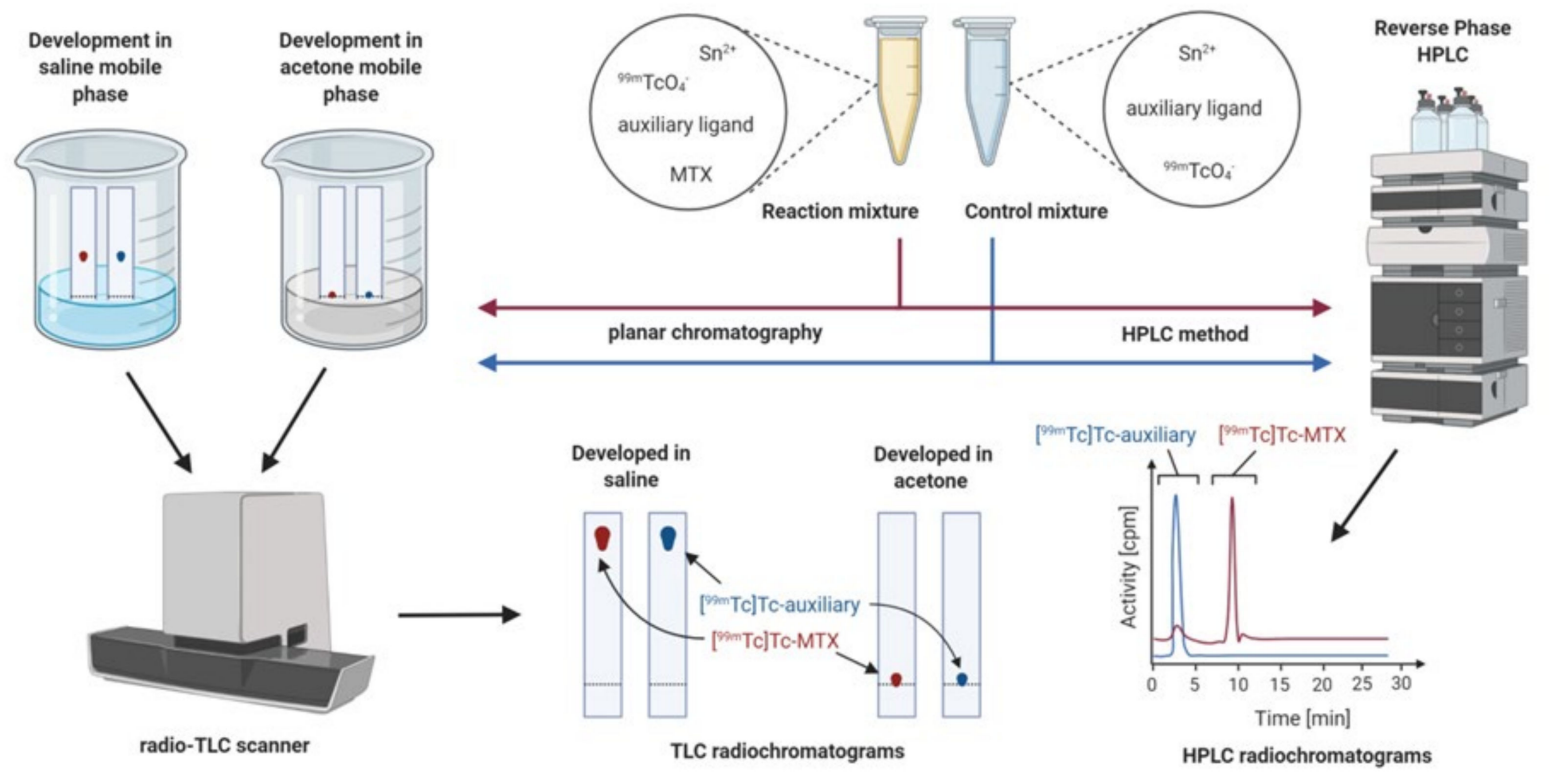
Schematic protocol of ITLC and HPLC comparative analyses executed within own studies on MTX labelling with ^99m^Tc.

Further on, we hypothesise that the final products of the MTX labelling reactions published in reports [5,6,7,8,9,10,11,12] were misinterpreted as final [^99m^Tc]Tc-MTX radiocomplex, while in reaction mixture they received the combination of the desired [^99m^Tc]Tc-MTX radiocomplex with one or more ^99m^Tc-intermediate complexes. In these cases, the logD value determined for aliquot from the reaction mixture is highly related with the yield of the MTX labelling reaction, more precisely, with the ratio of the ^99m^Tc-intermediate and the [^99m^Tc]Tc-MTX complexes in the reaction mixture. We determined experimentally in our study logP values for the ^99m^Tc-intermediate complexes and [^99m^Tc]Tc-MTX isolated previously from the reaction mixture using HPLC and for non-isolated [^99m^Tc]Tc-MTX (Table 5). For isolated [^99m^Tc]Tc-MTX radiocomplex logP is equal to −1.54 ± 0.05, while non-isolated radiocomplex mixture has lower logD value equal to −2.08 ± 0.05 (Table 5). Comparing these results with even lower values of −2.22 and −2.28 (Table 1) determined by Dar et al. [6] and Papachristou et al. [12], we conclude that authors determined their lipophilicity characteristics as the resultants of lipophilicity values of the individual reaction mixture components. Their logD values fall between desired [^99m^Tc]Tc-MTX radiocomplex logP value and the logP values of the ^99m^Tc-intermediate complexes (Table 5). This type of experiment should be verified in terms of radiocomplex stability during the experiment period through HPLC analysis. It is necessary to rely on HPLC method, that shows better resolution in the system, to determine whether the final desired complex [^99m^Tc]Tc-MTX has been formed in the reaction mixture properly and has not been decomposed during physicochemical studies (Figure 9).

## 3. Recommendations and Indications

All newly designed and synthesised radioagents should be precisely characterised, to provide the reliability of further biological activity research. This characterisation should include radiochemical yield, chemical purity, physicochemical characterisation as stability in conditions of interest, determination of lipophilicity and charge of the radioagent itself, and a description of the structure if it is possible to obtain an analogous reference compound [28]. In the case of radiopharmaceuticals containing ^99m^Tc (e.g., the [^99m^Tc]Tc-MTX radiopreparation discussed in this paper), the best way to their identify and structure determination is to obtain in a weight scale their analogues containing the long-lived ^99^Tc (t_1/2_ 4.2 × 10^6^ y) radionuclide or stable Re isotope. These so-called ‘cold reference compounds’ can be tested by standard physicochemical methods (elemental analysis of elements, mass spectroscopy, nuclear magnetic resonance spectroscopy, X-ray crystallography), which will give precise information about their composition and structure. However, in some cases, due to the high price and limited availability of Tc-99, as well as slightly different Re chemistry, this method of identification is not possible. Probably for this reason the structure of the [^99m^Tc]Tc-MTX radiocompound is still unknown (based on our own experience and literature data, we suppose that in the radiopreparation [^99m^Tc]Tc-MTX the technetium atom is coordinated by four carboxyl groups derived from two MTX molecules). In such cases, the other comparative identification methods must be particularly properly performed to ensure reliable findings.

### 3.1. Planar Chromatography Methods Validation in Radiopharmaceutical Chemistry

Planar chromatography techniques are simple, specific and accurate methods for quick qualitative assessment of the mixture composition. They are commonly applied in the nuclear medicine departments to evaluate the efficiency of radionuclide labelling reactions during the quality control of various radiopharmaceutical preparations. However, these techniques are incapable of qualitative identification of chemical compounds. They serve as analytical comparative methods and can only be used for referring to well-known systems. Application of these methods require prior evaluation of the appropriate analysis conditions, namely, selection of the stationary phase (e.g., paper chromatography, silica gel coated sheets) and the mobile phases (developing solutions) allowing for optimum separation of the mixture components. Under the designed analysis conditions, the retention factor values should be established for each reaction reagent separately. Additionally, R_f_ values should also be determined for main by-products or wastes (e.g., TcO_2_ colloids), if applicable. Final assessment of the reaction mixture composition through PC method bases on comparison of R_f_ values obtained for the tested sample with the R_f_ values previously determined for the individual reagents. 

Failure to comply with above rules often leads to misinterpretation of the experimental results, perfectly illustrated on the presented above MTX ^99m^Tc-labelling case study. Generally, during the ^99m^Tc labelling, [^99m^Tc]Tc^(VII)^O_4_^−^ anions are reduced and create a target ^99m^Tc complex, but also can be reduced further into [^99m^Tc]Tc^(IV)^O_2_ or be reoxidised into pertechnetate (by traces of O_2_). To assess the radiochemical purity of the obtained radiocomplex, two TLC analyses are performed in different mobile phases: organic (acetone, methyl-ethyl ketone or methyl alcohol) and aqueous (saline) (Figure 2). This assessment is performed mainly in a well-tested system as quantitative quality control of ^99m^Tc labelling efficiency and does not allow a qualitative determination of the products in the newly designed system.

### 3.2. Recommendations for Radiopreparation Stability Studies in Radiopharmaceutical Chemistry

The stability parameter must be carefully examined at the design and testing stage of a new potential radiopharmaceutical. Released radionuclides can distribute around the patient’s body, providing erroneous results of diagnostic imaging, and exposing healthy tissues on a redundant dose of radiation.

A measure of the strength of the interaction between radionuclide cation and applied chelator is called complex stability constant, *K* value, defined as an equilibrium constant of the complex formation reaction. The reaction of direct labelling with ^99m^Tc presented below is described by the association equation:

(1) T 99mcTcV+ligand →K T 99mcTcVligand 

where *K* is the complex formation constant.

(2) K=T 99mcTcVligand T 99mcTcV * ligand 

(3) T 99mcTcVligand =K * T 99mcTcV * ligand 

According to Le Chatelier’s principle, the excess of substrate ligand present in the reaction mixture stabilises the resulting ^99m^Tc-radiocomplex. Therefore, studies on the stability of not isolated radiocomplex from labelling reaction mixture are conducted incorrectly providing adulterated results. Unfortunately, in many works it can be found that stability study of newly designed radiopreparation has been evaluated as changes of the labelling reaction yield over time [29,30,31]. Moreover, from an ethical point of view, only radiopreparations that fully meet all requirements for radiopharmaceuticals may be allowed for studies on animals obligatory before clinical trials. This means that the determination of the physicochemical parameters of the tested radiopreparations should be performed in properly conducted experiments. 

We recommend to perform all challenge experiments, i.e., stability studies in solutions that mimic human blood (PBS, artificial cerebrospinal fluid, human serum) solutions containing high molar excess of competitive natural ligands containing reactive -SH or -NH_2_ groups, (histidine, cysteine, human serum), as well as biological studies (animal biodistribution) using radiocompound samples previously isolated from the reaction mixture and in imitating physiologic conditions (temperature, osmolarity).

### 3.3. Recommendations for Radiopreparation Lipophilicity Studies in Radiopharmaceutical Chemistry

Lipophilicity, defined as the decimal logarithm of the partition, P (of one molecular species), or distribution, D (of more than one molecular species), coefficient, is one of the most important physicochemical parameters that should be taken into account when developing new drugs, especially radiopharmaceuticals. It is significantly related with numerous pharmacokinetic properties such as drug absorption, distribution, physiological barrier permeation, and route of drug clearance. Lipophilicity value of new radiopreparation is determined usually in a biphasic system as the ratio of the radioactivity in the organic phase to the radioactivity in the aqueous phase. As a suitable and convenient system for lipophilicity evaluation accordingly mimicking the physiological conditions, it is recommended to use n-octanol and PBS buffer, pH 7.40, as the organic and aqueous phases, respectively (according to OPPTS, 830.7550, 1996). In practice, when performing a lipophilicity test, the sample of the isolated radiocompound of interest is introduced into the PBS phase. The sample volume should be as small as possible so as not to influence on the properties of the phase, or even fully evaporated. The use of a solution consisting of, e.g., 100 µL of the labelling reaction mixture and 200 µL of the aqueous phase [6] (Dar et al., 2012) does not meet the conditions recommended for performing a lipophilicity test. 

We highly recommend to perform lipophilicity determination for the pure radiopreparation isolated from reaction mixture. The presence of unbound radionuclide cations or radio-by-products can lead to incorrect determination of the D parameter. Likewise, the presence of other substances in the system (e.g., reducing agents, free ligands) may modify the phases and lead to obtain logD values with substantial errors. For very accurate lipophilicity measurements it is recommended to saturate both liquid phases with each other before use in the lipophilicity study system—this allows to eliminate errors resulting from the mutual solubility of both liquids. Furthermore, based on our own experience, we also recommend to perform radiopreparation lipophilicity studies with the HPLC control analysis of the aqueous phase after completing the radioactivity measurements.

**Figure 10:**
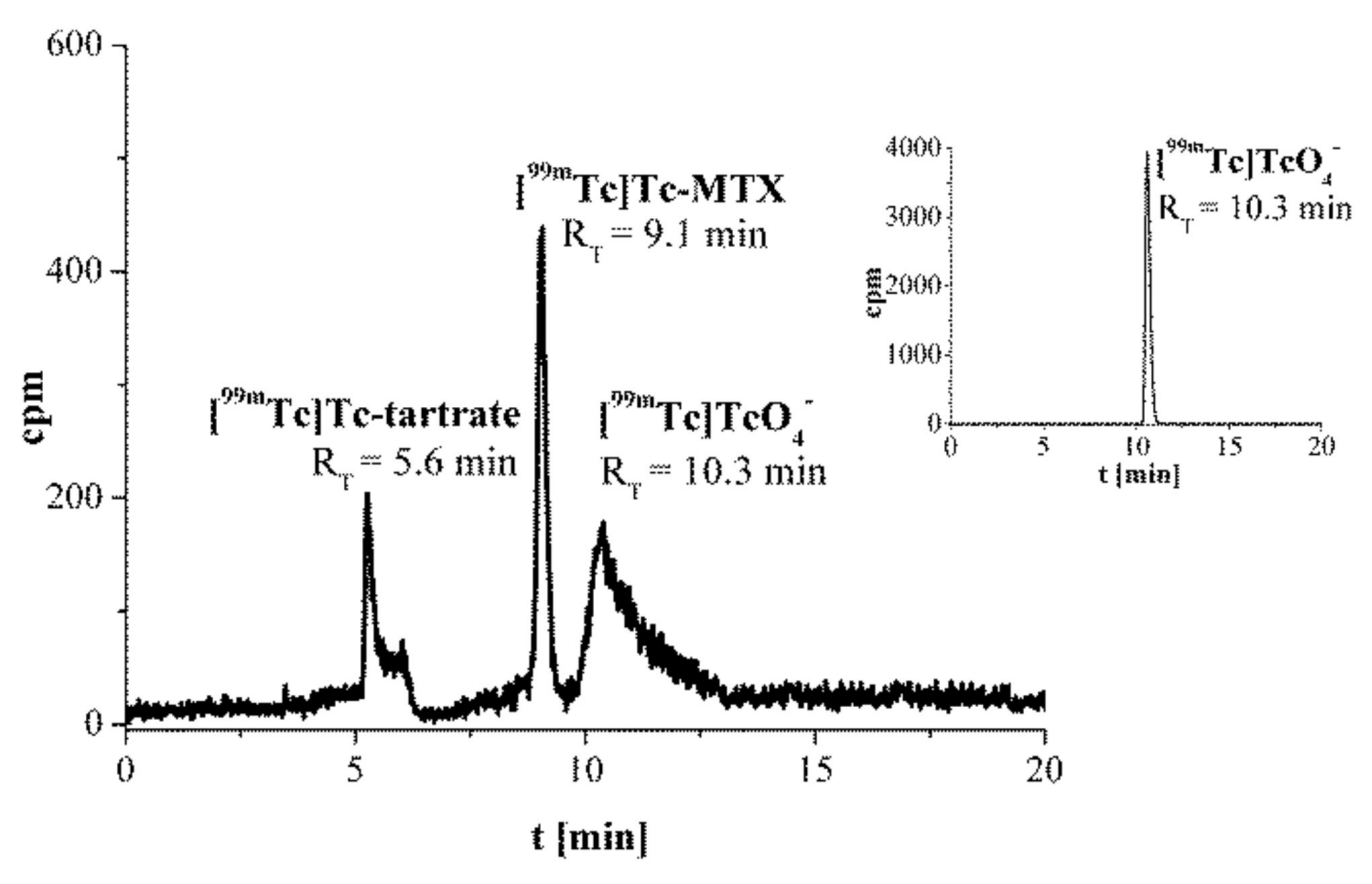
Radiochromatogram of aqueous phase of the [^99m^Tc]Tc-MTX reaction mixture recorded after lipophilicity test.

The logP value of isolated [^99m^Tc]Tc-MTX radiocomplex, determined in our experiments as mean value of three independent measurements done in duplicate, was equal to −1.54 ± 0.05 (Table 5). The HPLC radiochromatogram of aqueous phases after that lipophilicity study is presented in Figure 8. Simultaneously, determination of the logD parameter for the non-isolated [^99m^Tc]Tc-MTX radiocomplex provided a lower value of −2.08 ± 0.05 (Table 5). The HPLC radiochromatogram of the aqueous phase after lipophilicity studies in this case is presented in Figure 10. Three signals visible in this radiochromatogram with R_T_ at 5.6, 9.1 and 10.3 min correspond, respectively, to the intermediate [^99m^Tc]Tc-tartrate, [^99m^Tc]Tc-MTX radiocomplexes (Table 5) and [^99m^Tc]TcO_4_^−^ anion. The lipophilicity value obtained in this case is probably the resultant value of the logD parameters of the individual mentioned above mixture components and does not characterise the tested [^99m^Tc]Tc-MTX radiopreparation.

## 4. Materials and Methods

MTX was at least of analytical grade purity, purchased from TCI Europe and received as a kindly gift from National Medicines Institute. All other reagents and solvents were of analytical grade, purchased from Sigma-Aldrich, Merck, and used without further purification. Deionised water was prepared in a Hydrolab water purification system (Hydrolab, Straszyn, Poland). ^99m^Tc radionuclide was eluted from the commercially available ^99^Mo/^99m^Tc generator, (Institute of Atomic Energy, Radioisotope Centre POLATOM, Świerk-Otwock, Poland) as the [^99m^Tc]pertechnetate in 0.9% NaCl solution. Analyses, separation and purification processes were performed by the TLC and/or HPLC methods. 

Syntheses of [^99m^Tc]Tc-MTX complex were performed, in general, in labelling reaction using MTX, [^99m^Tc]NaTcO_4_ eluate from ^99^Mo/^99m^Tc generator, stannous chloride as reducing agent and in the presence or absence of various additional reagents (auxiliary ligands). Separate syntheses of the ^99m^Tc-intermediate complexes were carried out in parallel, each time using the same auxiliary ligand that was present in the reaction mixture during the synthesis of the [^99m^Tc]Tc-MTX radiocomplex. As more than 10 different labelling reactions were carried out in the work, and the obtained reaction results strongly depended on the composition of a given reaction mixture, the procedures for these syntheses are included in Section 2.3.1. along with the results, which will make it easier for the reader to follow the studied issues. 

TLC analysis conditions: TLC analyses were performed on ITLC-silica gel sheets using saline or acetone as developing solvents. The distribution of radioactivity on ITLC sheets was measured by Storage Phosphor System Cyclone Plus (Perkin-Elmer, Waltham, MA, USA) and analysed using OptiQuant software.

HPLC analysis conditions: Merck L7100 chromatographic set equipped with a UV-Vis detector (Shimadzu SPD-10AVP) and a homemade (INCT, Warsaw, Poland) radiometric well-type detector with the NaI(Tl) crystal. The separation was accomplished on Jupiter Proteo 90 Å, 4 µm, 10 × 250 mm HPLC semi-preparative column (Phenomenex, WA, USA); flow rate set as 2 mL/min with gradient elution: 20–80% A in 0–20 min, 80% A in 20–40 min; UV/Vis detection at 220 nm and/or γ-detection; where A—acetonitrile with 0.1 % (*v/v*) of trifluoroacetic acid and B—water with 0.1% of trifluoroacetic acid (*v/v*).

Lipophilicity study: 1 mL of PBS containing isolated radiocomplex was introduced to a test tube containing 1 mL of n-octanol. After vortexing for 1 min, the test tube was centrifuged for 5 min with 14,000 rpm to ensure complete separation of layers, then two 100 µL samples of each layer were collected. The activity of each layer sample was measured using the gamma counter. Partition coefficient P was calculated as the ratio of radioactivity of the organic to radioactivity of the aqueous phase. After measurements, HPLC analysis of the aqueous phase was performed to verify the stability of radiopreparation of interest. Lipophilicity was expressed as the decimal logarithm of the partition coefficient (logP), mean value of three independent experiments done in duplicate.

## 5. Conclusions

The radiopharmaceutical preparations synthesised in hospital nuclear medicine departments directly from kit systems for patient application are usually tested only by TLC or ITLC methods according to the kit producer validated procedure. These methods are the most commonly applied and recommended for determination of the potential radiochemical impurities of ^99m^Tc-radiopreparations: [^99m^Tc]TcO_4_^−^ and colloidal [^99m^Tc]Tc-oxides. However, final assessment of the synthesis progress in the case of novel radiopharmaceuticals (to identify them in the reaction mixture) must be validated at the stage of design and development of their syntheses using high resolution separation techniques, e.g., HPLC or GC.

The most common mistake at the stage of evaluation of any new radiopharmaceuticals is study of the physicochemical properties of new radiopreparations (stability, lipophilicity) from the reaction mixture samples. Many article authors provide even direct information that, e.g., the stability of the radiocompound was tested in the reaction mixture, sometimes there is no information on this matter at all, and there is hardly ever information that the tests were performed on the radiocompound previously isolated from the reaction mixture. Both stability and lipophilicity determination in inappropriate conditions influence parameters characterised for a given chemical compound, that is essential in the case of further clinical research and medicinal application. Failure to comply with the above rules can lead to misinterpretation of the experimental results.

## Figures and Tables

**Table 1:** Chromatography results and logD values of [^99m^Tc]Tc-MTX radiocomplexes obtained in the cited research articles.

Syntheses of [^99m^Tc]Tc-MTX (Additional Reagents)	PC R_f_ Values [Mobile Phase]		HPLC Analysis	logD	Reference
[^99m^Tc]Tc-MTX	At origin [acetone]	No data	No data	No data	[5]
[^99m^Tc]Tc-MTX (stannous tartrate, AA)	0.00–0.01 [acetone]	0.9–1.0 [saline]	No data	−2.22 *	[6]
[^99m^Tc]Tc-MTX	No data	No data	No data	No data	[7]
[^99m^Tc]Tc-MTX (stannous tartrate, AA, sodium citrate and pyrophosphate)	0.00–0.01 [acetone]	0.9–1.0 [saline]	No data	No data **	[8]
[^99m^Tc]Tc-MTX and [^99m^Tc]Tc-MTX-Glu_2_	At origin [acetone]	~1 [Pyr:AcOH:H_2_O 3:5:1.5]	No data	No data	[9]
[^99m^Tc]Tc-MTX and [^99m^Tc]Tc-MTX-Lys_2_	At origin [acetone]	~1 [Pyr:AcOH:H_2_O 3:5:1.5]	No data	No data	[10]
[^99m^Tc]Tc-MTX (AA and sodium citrate)	At origin [acetone]	~1 [saline]	No data	No data	[13]
[^99m^Tc]Tc-MTX (stannous tartrate and AA)	At origin [acetone]	~1 [ACN/ H_2_O /TFA; 50/25/1.5]	No data	No data	[11]
[^99m^Tc]Tc-MTX (sodium gluconate)	0.0–0.1 [acetone]	0.9–1.0 [saline]	Executed	−2.28 ± 0.03	[12]

* the logD values were calculated by the authors of this article based on the source data provided in the cited publication; ** authors in abstract provided that “Partition coefficient in n-octanol and saline indicated that the labelled radiopharmaceutical was hydrophilic”.

**Table 2:** Physicochemical parameters of the ^99m^Tc-complexes.

Syntheses of the ^99m^Tc-Complexes (Reducing Reagents)	PC R_f_ Values [Mobile Phase]		HPLC R_T_ [min]	logD	Reference
[^99m^Tc]Tc-EDTA (SnCl_2_)	At origin [acetone]	~1 [saline]	No data	No data	[15]
[^99m^Tc]Tc-citrate (SnCl_2_)	At origin [acetone]	~1 [saline]	No data	−2.46 *	[17]
			No data	No data	[18]
[^99m^Tc]Tc-AA (SnCl_2_)	0.0–0.15 [serum physiologic]	0.8–1.0 [ACD] **	1.04 ***	No data	[21]
[^99m^Tc]Tc-AA (FeCl_3_)	No data	No data	3.05 ****	No data	[22]
[^99m^Tc]Tc-α-hydroxy acids (SnCl_2_)	At origin [acetone]	~1 [saline]	No data	No data	[23]
[^99m^Tc]Tc-gluconate (SnCl_2_)	At origin [acetone]	~1 [saline]	No data	No data	[24]

* logD value was calculated by the authors of this work based on the source data (radiocomplex partition in biphasic system saline/n-butanol) provided in the cited publication; ** ACD: 0.068 M citric acid/0.074 M dextrose, 1:1 (*v/v*) at pH 5; *** HPLC conditions: analytical RP-C18 column (Macherey-Nagel, EC150/4.6 Nucleosil 100-3C-18); flow rate: 1 mL/min, mobile phase: 25 mM KH_2_PO_4_ (pH 3)/methanol, 96:4 (*v/v*). **** HPLC conditions: Nucleosil column with the guard column (Hichrom, NC100-5C18); flow rate: 1 mL/min, mobile phase: 25 mM K_2_HPO_4_ (pH 3)/methanol, 96:4 (*v/v*); UV detection wavelength 210 nm.

**Table 3:** Radiochemical yield of [^99m^Tc]Tc-tartrate intermediate complex relative to the amount of potassium tartrate.

Potassium Tartrate [mg]	RCY [%]
0.22	15
0.44	60
0.66	88
0.88	90
1.1	>95
1.32	>95

**Table 4:** Radiolabelling yield of [^99m^Tc]Tc-MTX radiocomplex relative to the amount of MTX and time.

Potassium Tartrate [mg]	MTX [mg]	RCY in 5 min [%]	RCY in 120 min [%]
1.1	1	25	59
1.1	2.5	27	72
1.1	5	41	92
1.1	10	44	87

**Table 5:** Determined physicochemical parameters of the ^99m^Tc-complexes.

^99m^Tc-Radiocomplex	PC R_f_ Values		HPLC R_T_ [min]	logP
	Acetone	Saline		
[^99m^Tc]Tc-AA	~0	~1	5.8	−3.08 ± 0.09
[^99m^Tc]Tc-EDTA	~0	~1	5.4	−3.99 ± 0.11
[^99m^Tc]Tc-gluconate	~0	~1	5.1	−4.05 ± 0.07
[^99m^Tc]Tc-tartrate	~0	~1	5.6	−2.46 ± 0.03
[^99m^Tc]Tc-MTX (not isolated)	~0	~1	9.1	−2.08 ± 0.05 *
[^99m^Tc]Tc-MTX (isolated)	~0	~1	9.1	−1.54 ± 0.05

* For the aliquot from the reaction mixture of the [^99m^Tc]Tc-MTX radiocomplex, lipophilicity should be specified as logarithm of the distribution coefficient, logD value.

## Abbreviations

AA	ascorbic acid
EDTA	ethylenediaminetetraacetic acid
FA	folic acid
Glu	glutamic acid
ITLC	instant thin layer chromatography
Lys	lysine
MTX	methotrexate
PBS	phosphate-buffered saline
R_f_	retention factor
R_T_	retention time
TLC	thin layer chromatography
